# Aldehyde Dehydrogenase Deficiency as Cause of Facial Flushing Reaction to Alcohol in Japanese

**Published:** 1995

**Authors:** David Goldman

**Affiliations:** David Goldman, M.D., is director of the Laboratory of Neurogenetics in the Division of Intramural Clinical and Biological Research at the National Institute on Alcohol Abuse and Alcoholism, Bethesda, Maryland

**Keywords:** aldehyde dehydrogenase, alcohol flush reaction, Asian, metabolism, acetaldehyde, genetics and heredity, AOD dependence

**Figure:**
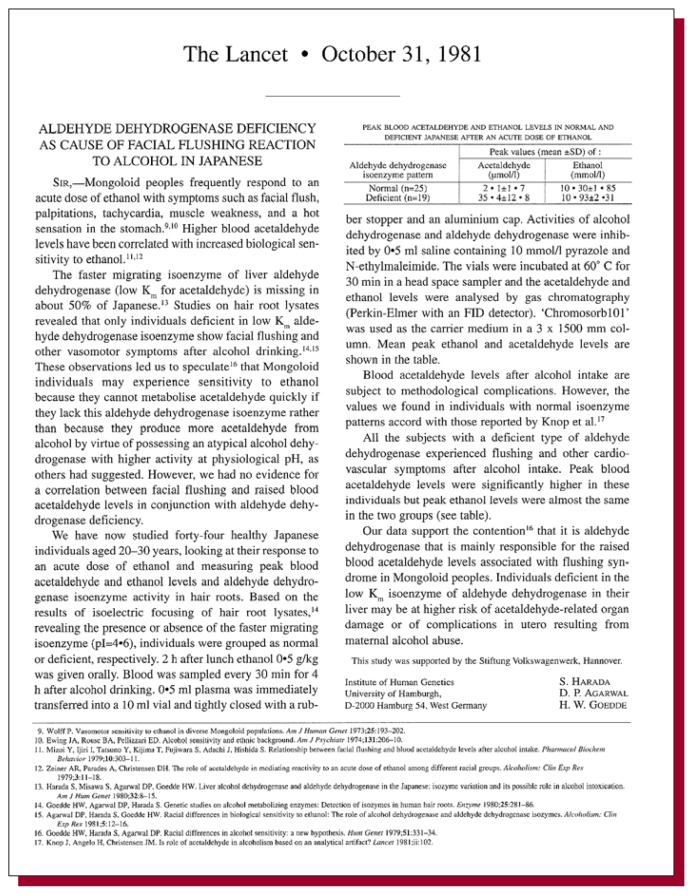
Harada, S.; Agarwal, D.P.; and Goedde, H.W. Aldehyde dehydrogenase deficiency as cause of facial flushing reaction to alcohol in Japanese. *Lancet* ii(8253):982, 1981.

Aldehyde dehydrogenase is an enzyme responsible for the breakdown of acetaldehyde, a toxic intermediate^1^ produced directly from the metabolism of alcohol. Aldehyde dehydrogenase is extraordinarily efficient in keeping acetaldehyde levels very low, even immediately after alcohol consumption when acetaldehyde is being generated rapidly in the liver.

Multiple aldehyde dehydrogenase enzymes exist and their structures are determined by different genes; however, this commentary examines only the enzyme (designated as ALDH2) that is imported into the mitochondrion, the cell’s energy-producing structure. The seminal article by Harada and colleagues, which actually is a detailed letter to the editor of the journal *Lancet*, first related the discovery of the effect of a genetic variant of this enzyme, which occurs very abundantly in Oriental populations (East Asians).

ALDH2 is key in the metabolism of acetaldehyde produced after alcohol consumption. In 1948 Hald and Jacobsen introduced disulfiram (Antabuse^®^), an inhibitor of aldehyde dehydrogenase, for the clinical treatment of alcoholics. Disulfiram-treated subjects who consume alcohol accumulate relatively high levels of acetaldehyde. These high acetaldehyde levels lead to an aversive response known as the flushing reaction. In addition to a facial flush, the flushing reaction also may include an elevated heart rate (i.e., tachycardia), headache, heart palpitations, shortness of breath (i.e., dyspnea), hyperventilation, low blood pressure (i.e., hypotension), vertigo, nausea, and vomiting. Certain other drugs that also inhibit aldehyde dehydrogenase, such as metronidazole, were found to cause flushing in association with alcohol intake. This flushing reaction also occurs naturally in some people. However, the blockade of aldehyde dehydrogenase actually was used to treat alcoholism long before the mechanism that causes naturally occurring alcohol-induced flushing was discovered.

Harada and colleagues directly related naturally occurring aldehyde dehydrogenase blockade to elevated acetaldehyde levels and flushing. Wolff (1972) and others (Zeiner et al. 1979) had observed that unmedicated Japanese and people from neighboring regions of Asia often showed the same flushing response after intake of relatively small quantities of alcohol. In 1981 Harada and colleagues Agarwal and Goedde showed that some people were naturally deficient in the aldehyde dehydrogenase enzyme (Agarwal et al. 1981). By using a technique (i.e., protein electrophoresis) that separates the enzyme in an electric field, followed by a stain for the enzyme, they found that acetaldehyde dehydrogenase was structurally different in people in whom the aldehyde dehydrogenase activity was deficient. One year later these researchers published their seminal letter to the editor of *Lancet*, relating findings that closed the link between the aldehyde dehydrogenase enzyme deficiency and alcohol-induced flushing. People with the deficient aldehyde dehydrogenase were clearly shown to be those with high acetaldehyde levels after alcohol consumption.

A cascade of research was triggered by the observations Harada and colleagues reported in this letter. The precise demonstration that the functional difference in alcohol metabolism in people with East Asian ancestry was attributable to a structural difference in ALDH2 led to studies of the enzyme protein and of the gene that determines this enzyme. It could be said that, for alcoholism, the molecule of the 1980’s was aldehyde dehydrogenase. Two years after this letter appeared, Yoshida and colleagues (1984) showed that the cause of ALDH2 inactivation in East Asian subjects was a substitution of a single amino acid (i.e., lysine substituted for glutamate in position 487 of the protein chain). This amino acid substitution was itself found to result from a single DNA base substitution among the thousands of DNA bases that make up the ALDH2 gene. Structurally, the aldehyde dehydrogenase enzyme is composed of four usually identical subunits (i.e., it is a tetramer). The tetramer was found to be inactivated if even one of its four subunits had the glutamate 487 substitution. It is for this reason that the inactive variant of the gene, designated ALDH2^2^, follows a dominant pattern of inheritance. For example, if the version of ALDH2 inherited from either one of an individual’s parents is ALDH2^2^, there is nearly a complete loss of aldehyde dehydrogenase enzyme activity.

Gene-geography studies revealed that aldehyde dehydrogenase deficiency was highest in people from East Asia and absent or nearly absent in whites and blacks. DNA-based tests showed that the DNA substitution responsible for the deficiency was the same across populations and also within populations in which the deficiency is abundant. Epidemiological studies of populations with a high prevalence of ALDH2^2^ revealed that aldehyde dehydrogenase deficiency dramatically lowers vulnerability to alcoholism. In doing so, ALDH2 deficiency interacts with other factors, including a common, superactive genetic variant of alcohol dehydrogenase (ADH2^2^) which increases the rate that acetaldehyde is produced. Thus, a single DNA nucleotide difference, present in hundreds of millions of people, profoundly influences their experiences with alcohol and vulnerability to alcoholism.

A mark of good scientific paradigms is their propensity for shaping the future by posing answerable questions that open new intellectual vistas. For aldehyde dehydrogenase, many of the most interesting and humanistically most significant questions remain unanswered. For example, what are the additional risks besides flushing for people with aldehyde dehydrogenase deficiency who consume alcohol? This question is being intensively explored in several ways, including comparing alcoholics with and without liver disease.

Researchers are only beginning to ask other important questions. For example, why do East Asians so frequently have aldehyde dehydrogenase deficiency—by chance or necessity (i.e., was there some biological [selective] advantage in ancient times and is there any current advantage to possessing the ALDH2^2^ gene)? Do people who abstain from alcohol use suffer any negative consequences of aldehyde dehydrogenase deficiency?

Much of the current excitement in alcohol research revolves around identifying genetic factors that, like ALDH2^2^, act to influence a person’s vulnerability to alcohol but that act at the level of the brain. Such factors may lead a person to seek alcohol more avidly and respond to it with greater or lessor sensitivity. Intensive family studies, such as the National Institute on Alcohol Abuse and Alcoholism’s Collaborative Study on Genetics of Alcoholism (COGA), are underway to identify such factors. Once the genes are identified, gene-environment and gene-gene interaction studies will be required to better understand the forces that combine in the development of vulnerability for alcoholism. The epidemiological studies on ALDH2^2^ show that such genetic factors act probabilistically rather than deterministically to influence alcoholism vulnerability. In other words, people with a genetic vulnerability factor may have a higher probability of becoming alcoholics, but they are not predestined to developing the disease.

What are the factors that influence some individuals to become alcoholics, even though they have aldehyde dehydrogenase deficiency? Studies of alcoholics who carry the protective aldehyde dehydrogenase variant may reveal other genetic and nongenetic factors that lead to alcoholism or that are protective for alcoholism. Examples of gene-gene and gene-environmental influences involving aldehyde dehydrogenase include the finding that variations in alcohol dehydrogenase play an interacting role in alcoholism vulnerability (Thomasson et al. 1991) and the discovery that vulnerability to alcoholism is increased in East Asians who have immigrated to North America.

## Footnotes

1 A chemical compound formed as an intermediate step between the initial form (i.e., alcohol) and the final product (i.e., acetate).
